# Supplementary figures and images for: A potential mechanism for tetraspanin CD82-mediated regulation of EGFR
Source: Life Sci Alliance. 2026 May 13;9(7):e202503426. doi: 10.26508/lsa.202503426 (PMC13171294; doi:10.26508/lsa.202503426)

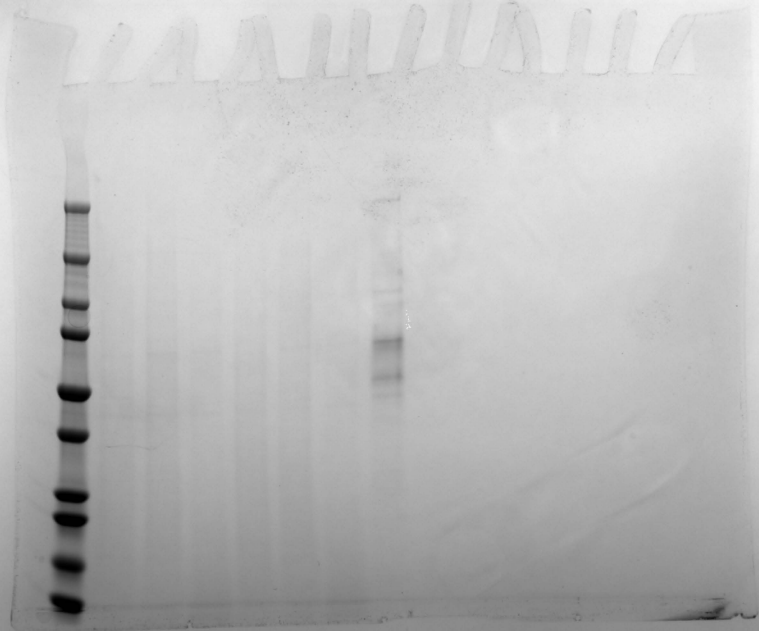

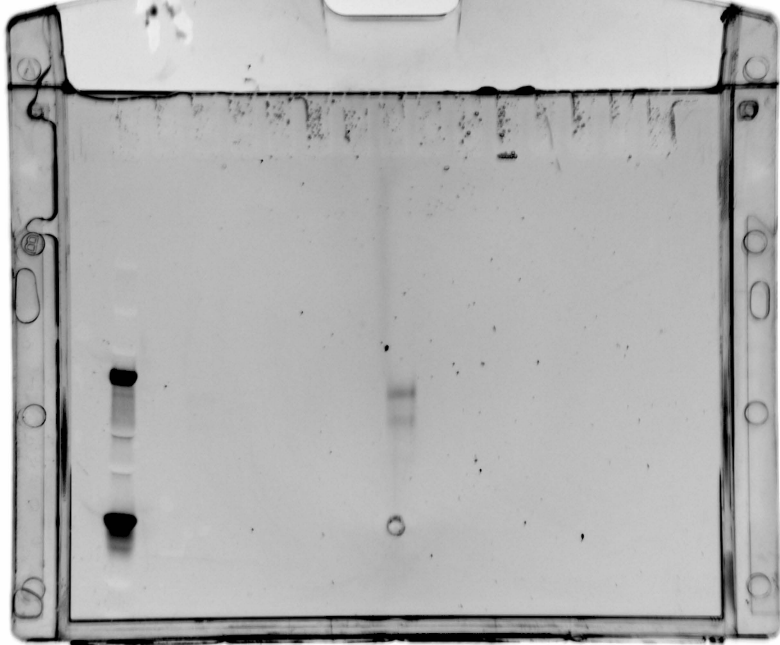

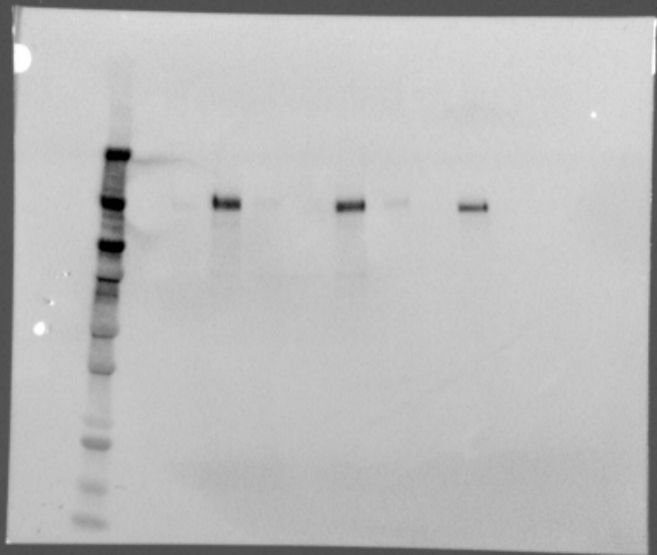

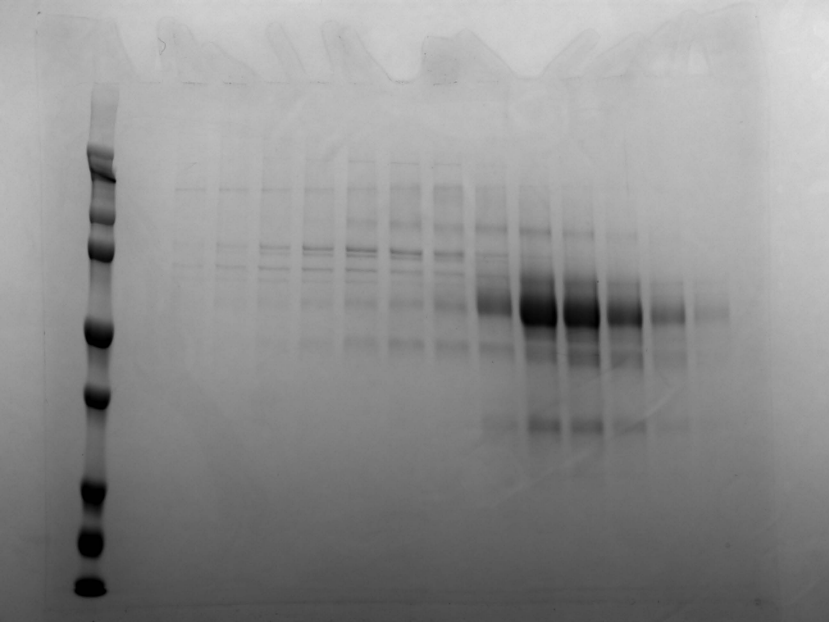

Supplement: Supplementary file 1 [file LSA-2025-03426_SdataF1.1.pdf]

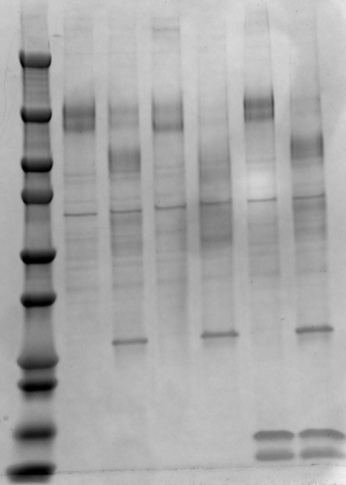

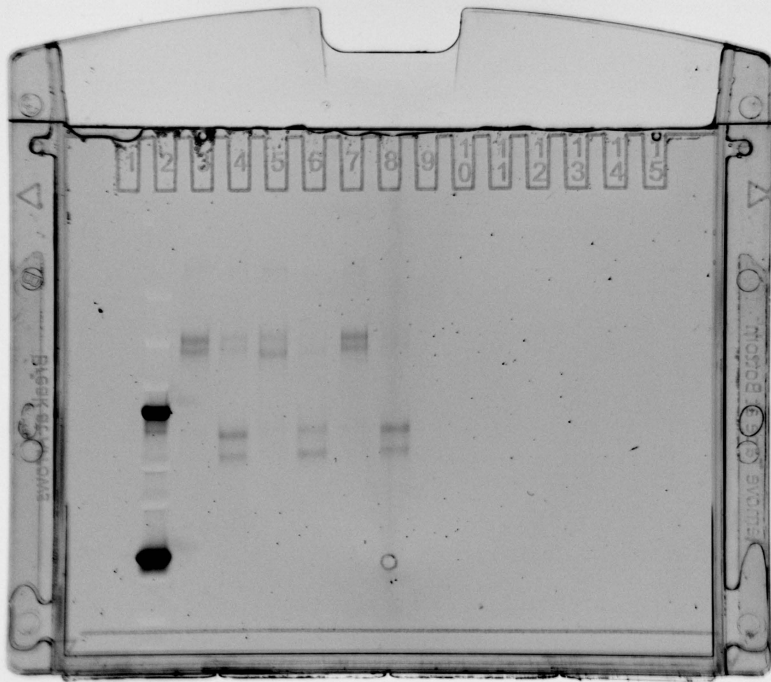

Supplement: Supplementary file 3 [file LSA-2025-03426_SdataF2.1_F4.1.pdf]
